# Chemical Hydrogels Bearing Thiazolium Groups with a Broad Spectrum of Antimicrobial Behavior

**DOI:** 10.3390/polym12122853

**Published:** 2020-11-29

**Authors:** Alexandra Muñoz-Bonilla, Jakub Zagora, Daniela Plachá, Coro Echeverría, Alberto Chiloeches, Marta Fernández-García

**Affiliations:** 1 Instituto de Ciencia y Tecnología de Polímeros (ICTP-CSIC), C/Juan de la Cierva 3, 28006 Madrid, Spain; sbonilla@ictp.csic.es (A.M.-B.); cecheverria@ictp.csic.es (C.E.); achiloeches@ictp.csic.es (A.C.); 2 Interdisciplinary Platform for Sustainable Plastics towards a Circular Economy-Spanish National Research Council (SusPlast-CSIC), 28006 Madrid, Spain; 3 Nanotechnology Centre, VŠB–Technical University of Ostrava, 15. Listopadu 2172/15, 70800 Ostrava-Poruba, Czech Republic; jakub.zagora@vsb.cz; 4 Center of Advanced Innovation Technologies, VŠB–Technical University of Ostrava, 15. listopadu 2172/15, 70800 Ostrava-Poruba, Czech Republic; 5 Centre ENET, VŠB–Technical University of Ostrava, 17. listopadu 2172/15, 708 00 Ostrava-Poruba, Czech Republic

**Keywords:** hydrogel, thiazole, quaternization, antimicrobial

## Abstract

Several hydrogels based on 2-hydroxyethyl methacrylate and a methacrylic monomer containing a thiazole group in its lateral chain have been prepared by thermal polymerization at 60 °C in water solution varying the chemical composition of the gels. The posterior quaternization of the thiazole groups with methyl iodine has rendered positively charged hydrogels with potential antimicrobial activity. This modification has been structurally characterized by infrared spectroscopy, whereas the thermal stability of all hydrogels has been studied by thermal degradation in inert atmosphere. The swelling behavior in distilled water and the rheology of the different hydrogels have been analyzed as a function of 2-(4-methylthiazol-5-yl)ethyl methacrylate (MTA) monomer content as well as its methylation. Finally, the active character of hydrogels against Gram-positive and Gram-negative bacteria and fungi has been evaluated, revealing excellent antimicrobial activity against all tested microorganisms. The methylated hydrogels could be used as potential materials for wound healing or contact lens applications.

## 1. Introduction

Polymeric biomaterials are used in many applications, such as medical implants and devices, drug delivery, wound dressings, sutures, etc. [1,2,3,4,5,6], and hydrogels represent a great percentage of these materials. Due to the physical or chemical crosslinked polymeric networks, hydrogels can absorb substantial large amount of water compared to its dry state and may also have many different forms, including particles, capsules, membranes, films, or coatings. In addition to the advantage of absorbing and retaining moisture, hydrogels can load and release biomolecules and drugs, and are typically employed as drug delivery systems. Likewise, bioactive components can be integrated in the hydrogel structures providing this bioactivity. Chemical modifications of hydrogels as well as the use of bioactive monomers in the preparation processes are the two main approaches to fabricate inherent bioactive hydrogels. For instance, several investigations have been focused on the preparation of systems with antimicrobial activity [7,8,9,10,11,12,13,14,15,16], which make them especially useful to avoid or minimize the risk of infections. In the case of delivery systems, the activity of these materials frequently does not continue during time. To extend the durability and also to avoid the possible antimicrobial resistance to these drugs, the incorporation of polymers with antimicrobial activity to hydrogels is one of the best strategies to achieve surfaces with high efficiency against microorganisms. As is well-described, there are different parameters that influence the activity of antimicrobial polymers, such as molecular weight, hydrophilic/hydrophobic balance, charge density, electrostatic and hydrophobic interactions, and nature of counter-ions (either cations or anions) [17,18,19,20,21]. Despite these parameters, in the case of hydrogels the type and concentration of crosslinker may also affect the antimicrobial character [22,23]. 

In recent years, our group has been working in the development of polymeric materials with antimicrobial properties, mainly based on macromolecular structures containing cationic thiazolium groups in the lateral chain. Thiazolium is a naturally bioactive component of thiamine (vitamin B1) with proven antimalarial and antimicrobial properties and low toxicity [24,25,26,27]. The polymeric systems bearing thiazolium groups developed by our group also results in very highly efficient systems against a spectrum of microorganisms. A variety of polymers has been prepared including homopolymers, statistical and block copolymers synthesized by conventional or controlled radical polymerization [28,29,30,31,32]. Then, some statistical and block copolymers have been also used as additives in polymer blends to obtain films, coatings or fibers [33,34,35,36]. However, their structuration as hydrogels has not been yet tested. Consequently, in the present work we have synthesized different chemical hydrogels based on methacrylic monomers, 2-hydroxyethyl methacrylate (HEMA) commonly used in biomedical applications as exhibits low toxicity, and biocompatibility [1], and 2-(4-methylthiazol-5-yl)ethyl methacrylate (MTA) which also demonstrates very low cytotoxicity against human [26]. Then, these hydrogels have been cationically charged by alkylation reaction of the thiazole groups. The effects of MTA content and the alkyl modification on the structure, thermal, rheological and swelling properties have been widely analyzed. Finally, these parameters have been also studied on the hydrogel antimicrobial effectiveness against bacteria and fungi.

## 2. Materials and Methods 

### 2.1. Materials

5-(2-hydroxyethyl)-4-methylthiazole (98%, Sigma Aldrich, Darmstadt, Germany), 4-dimethylaminopyridine (DMAP) (99%, Sigma Aldrich), 2-methylpropenoic acid (99%, Sigma Aldrich), *N*,*N*′-dicyclohexyl carbodiimide (DDC) (99%, Sigma Aldrich), 4-(2-(methacryloyloxy)-ethoxy)-4-oxobutanoic acid (99%, Sigma Aldrich), anhydrous acetonitrile (CH_3_CN) (99.8%, Alfa Aesar), 2-hydroxyethyl methacrylate (HEMA) (99.99%, Sigma-Aldrich), poly(ethylene glycol) diacrylate (*M*_n_ = 575 g/mol, PEGDA) (99.99%, Sigma-Aldrich), 4,4’-azobis (4-cyanopentanoic acid) (ACPA) (≥75%, Sigma-Aldrich), phosphate buffered saline powder (PBS, pH 7.4, Sigma Aldrich) and NaCl solution (0.9%, BioXtra, suitable for cell culture, Sigma Aldrich) were used as received.

### 2.2. Synthesis of Hydrogels

MTA monomer was prepared as reported in literature [28]. Briefly, a solution of 5-(2-hydroxyethyl)-4-methylthiazole (40.9 mL, 350 mmol), DMAP (4.2 g, 35.0 mmol), distilled 2-methylpropenoic acid (38.4 mL, 454 mmol) in anhydrous CH_3_CN (175 mL) was prepared. This solution was added to a solution of DCC (94.0 g, 454 mmol) in anhydrous CH_3_CN (225 mL) previously prepared in a two neck round bottom flask equipped with a pressure-equalizing dropping funnel. The mixture was stirred under argon atmosphere and left for 24 h at 0 °C. After filtration, yellow oil was obtained.

Hydrogels were prepared from HEMA and MTA monomers using four different molar ratios via conventional radical polymerization in aqueous solutions with ACPA as radical initiator and PEGDA as crosslinking agent. The weight percentage of each component is gathered in Table 1. Briefly, the mixture of components was poured into a 5.0 cm × 5.0 cm × 0.1 cm template consisting of two glasses and a piece of rubber in-between. The samples were secured with clamps and placed in an oven at 60 °C for 3 h. After that, each sample was placed in deionized water and after 20 min were removed from the glass template and left in water for 2 h. The last step was cutting into pieces of 2 and 0.6 cm diameters for physicochemical characterization and antimicrobial activity, respectively. The prepared hydrogels were denoted as HG0, HG5, HG10 and HG20 depending of the MTA content related to HEMA (MTA/HEMA).

Subsequently, the corresponding cationic hydrogels with different positive charge at equilibrium were prepared by N-methylation of thiazole groups of the MTA units contained in the hydrogels. The N-methylation was carried out with methyl iodide in ethanol. The procedure was made as follows: hydrogel samples in cylindrical shape were put into 100 mL bottles. Different volume of methyl iodide (from 0.5 to 2.2 mL according to MTA content in each hydrogel) and 50 mL of ethanol were added. Thus, the reaction was carried out with an excess of the alkylating agent at room temperature to ensure the complete quaternization. Then, the reaction was stopped, and the methylated samples were washed repeatedly with ethanol to remove residual unreacted methyl iodide. The methylated forms of hydrogels were denoted as HG5Me, HG10Me and HG20Me.

### 2.3. Characterization Techniques

The chemical structures of hydrogel samples were analyzed using the attenuated total reflectance Fourier Transformation infrared spectroscopy (ATR-FTIR) with a VERTEX 70v Bruker instrument operating in the region of 4000–500 cm^−1^ with a resolution of 4 cm^−1^. Thermogravimetric analyses were performed using TGA/STA-FTIR-MS, in a SETARAM instrument from ambient to 800 °C with temperature ramp of 10 °C/min under the argon atmosphere. 

The rheological tests were performed using a Rheometer AR 1000 equipped with a Peltier device for temperature control. The experiments were performed using plate–plate geometry (diameter 20 mm) at 25 °C. The following tests were performed: (i) Torque sweep test in the range of 0.01–10,000 microN·m at a constant non-destructive frequency of 1 Hz in order to determine the Linear Viscoelastic Region (LVR); (ii) frequency sweep tests in the range of 100 to 1 Hz, performed within the LVR. Each system was measured a minimum of five times with different samples and the represented curves are the average of all. 

The TECATOR 6110 balance was used to evaluate the swelling capacity of hydrogels through measurements of the changes of hydrogels weight after their immersion in distilled water in closed test tubes for selected period. The prepared dried hydrogels were weighed and then immersed in distilled water at room temperature. Therefore, at selected time intervals, each sample was withdrawn from solution, an excess of solution on the sample surface was removed with a filter paper and sample was weighed. This process of weighting was repeated until the weight reached an equilibrium state. The follow equation for determining of swelling ratio values W_SW_ was applied:

W_SW_ = (W_t_ − W_0_)/W_0_ × 100 (1)

where W_t_ is the sample weight swelled at time t and W_0_ the weight of dry hydrogel.

For the microbiological assays, sheep blood (5%) Columbia agar plates were purchased from bioMérieux. American Type Culture Collection (ATCC): Gram-negative *Escherichia coli* (*E. coli*, ATCC 25922) *Pseudomonas aeruginosa* (*P. aeruginosa*, ATCC 27853), and Gram-positive *Staphylococcus aureus* (*S. aureus*, ATCC 29213), *Staphylococcus epidermidis* (*S. epidermidis*, ATCC 12228) bacteria and *Candida parapsilosis* (*C. parapsilosis*, ATCC 22019) yeast were obtained from Oxoid^TM^. Microorganisms were incubated for 24 h for bacteria and 48 h for yeast at 37 °C in a Jouan IQ050 incubator. The optical density of the microorganism suspensions was measured in McFarland units proportional to microorganism concentration by a DensiCHEK™ Plus (VITEK, bioMérieux). The bacteria suspensions of about 10^8^ colony-forming units (CFU) were prepared by adjusting concentration with saline solution to ca. 0.5 McFarland turbidity standard. Suspensions of ca. 5 × 10^5^ and 5 × 10^3^ CFU/mL for bacteria and fungi, respectively, were finally obtained by further dilution with PBS. The antimicrobial activity of the prepared hydrogels was determined following the E2149-13a standard method of the American Society for Testing and Materials (ASTM)[37]. Each hydrogel was placed in a sterile falcon tube and then, the bacterial suspension was added. Falcon tubes with only the inoculum were prepared as control experiments. The samples were shaken at room temperature at 150 rpm for 24 h. Bacterial concentrations at time 0 and after 24 h were determined by the plate counting method. Each sample was measured twice and counted at least by duplicate. The error bars are given as standard deviation. 

## 3. Results

**Figure 1:**
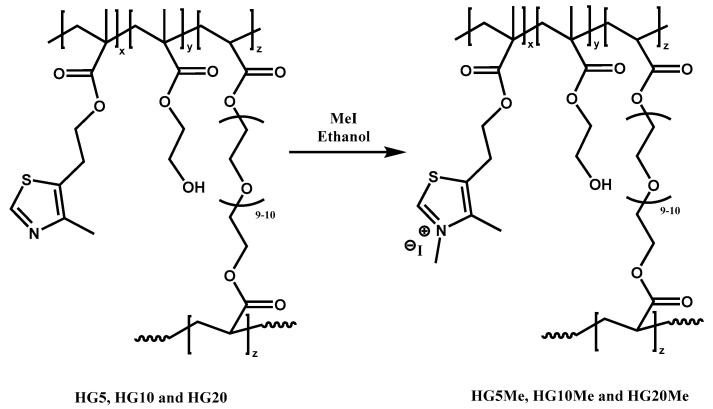
Chemical structure of the unmodified and cationic hydrogels obtained by quaternization reaction.

Hydrogels of HEMA, MTA and PEGDA were prepared by conventional radical polymerization using a 2 wt% of ACPA with respect to the total of HEMA and PEGDA monomers. The ratio between HEMA and PEGDA was kept constant at 5 and then, different amounts of active MTA monomer were introduced as specified in Table 1. The posterior methylation was performed in ethanol with methyl iodide at room temperature (Figure 1). 

**Figure 2:**
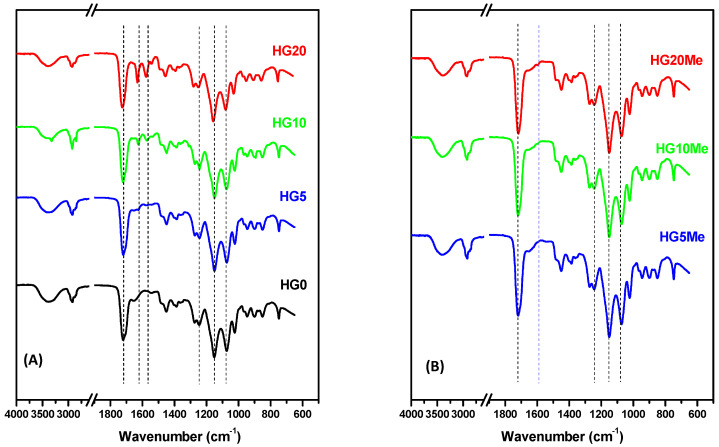
Attenuated total reflectance Fourier Transformation infrared spectroscopy (ATR−FTIR) spectra of (**A**) unmodified and (**B**) methylated hydrogels.

Figure 2 shows the corresponding ATR-FTIR spectra of (A) unmodified hydrogels and (B) after methylation. The carbonyl stretching vibration (C=O) at 1716–1722 cm^−1^ is visible in all spectra of unmodified and methylated hydrogels and corresponds to carbonyl groups present in monomers as well as in the crosslinker. The O–H stretching region around 3700–3100 cm^−1^ and the peaks between 1300 and 1000 cm^−1^ associated to the group O–C–O which are typical for HEMA polymers [38]. In the samples containing MTA units, spectra show the vibration band at 1626 cm^−1^ assigned to ʋ(C=C) of vinyl in thiazole, and at 1573 cm^−1^ the vibration of C=N bond, both corresponding to the thiazole group. These bands increase in intensity as MTA percentage in the hydrogel formulation augments and more evident in sample HG20, with higher percentage of MTA. The quaternization reaction leading the cationic hydrogels produces a change in the structure of hydrogel, the vibration of C=N bond corresponding to the thiazole group at 1573 cm^−1^ disappears and a new band emerged around 1595 cm^−1^, characteristic of the C=N^+^– stretching vibration, whereas the C=C band shifts to 1650 cm^−1^.

**Figure 3:**
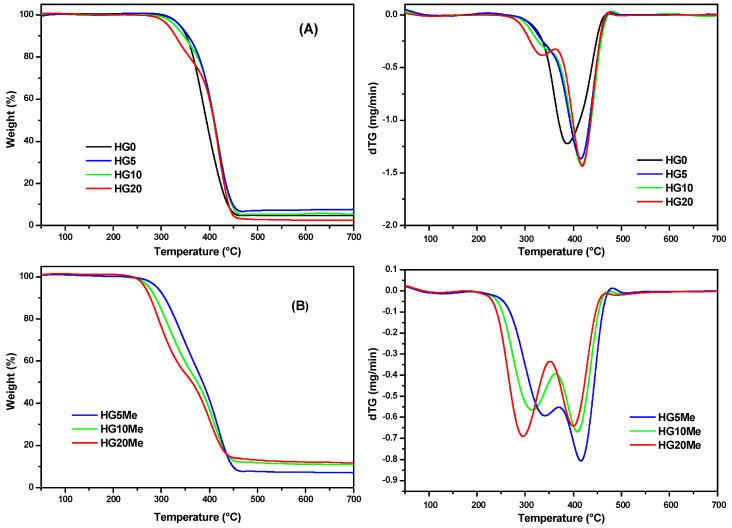
Thermogravimetric analysis (TGA) and dTG curves of (**A**) unmodified and (**B**) methylated hydrogels.

The variation of the ratio MTA/HEMA in the hydrogel was also analyzed by studying the thermal stability behavior under inert atmosphere at a heating rate of 10 °C/min. Figure 3 shows the thermogravimetric curves (TG) and their corresponding derivative (dTG) of (A) unmodified hydrogels and (B) after methylation. The characteristics parameters of degradation, the onset degradation temperature (*T*_0_), the maximum degradation temperature (*T*_max_) and the residue values are collected in Table 2.

The thermal degradation of the HG0 based only on HEMA takes place in only one step. During the heating, depolymerization occurs leading molecules of HEMA monomer and PEGDA as probably degradation products [38,39,40,41]. In case of the unmodified hydrogels containing different amounts of MTA monomer, the degradation occurs in two steps and is dependent on MTA/HEMA ratio. The stability of hydrogels decreases with the increase of MTA monomer [32]. After quaternization, the resulting hydrogels are less stable and there is a larger residue in comparison with the unmodified hydrogels.

**Figure 4:**
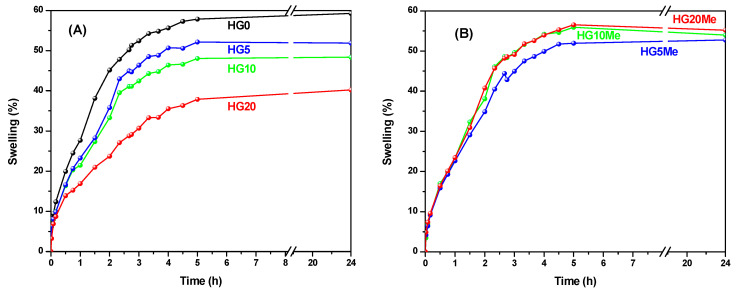
Swelling behavior in water of (**A**) unmodified and (**B**) methylated hydrogels.

Swelling properties are important characteristics of hydrogels and crucial for their applications; thus, their swelling capacities were studied by gravimetric analysis in water. Figure 4 displays the behavior of (A) unmodified and (B) quaternized hydrogels.

As can be observed, HG0 presents the highest swelling capacity of all unmodified hydrogels [42]. As hydrophobic MTA units are incorporated in the hydrogel, the capacity of water absorption significantly decreases. On the other hand, the posterior methylation produces an increase of the hydrophilic character of the MTA units and thereby of the hydrogels, with an increase of swelling capacity, which is somehow constrained by the network. The behavior of HG5Me is quite similar to HG5, but in the case of HG10Me and HG20Me, the differences are larger, almost reaching the behavior of HG0. Even if the increase of MTA content and further methylation ends up in an increased hydrophilic character, the swelling is also constrained by the network composition. This could mean that the swelling ability is a compromise between the increased hydrophilicity due to methylated MTA and the crosslinker degree of the hydrogel itself that limits the network’s maximum equilibrium swelling.

Further characterization of the hydrogels’ properties was performed by rheological studies. The rheological behavior of hydrogels was performed for the hydrogels swollen to the equilibrium state. Initially, the linear viscoelastic region (LVR), defined as the region where the values of G’ and G” are independent of the applied torque, was determined for each formulation through a torque sweep test in the range of 0.01–10000 microN·m at a non-destructive frequency of 1.0 Hz. Further, frequency sweep test in the range of 0.1-100 Hz were performed within the previously determined LVR.

**Figure 5:**
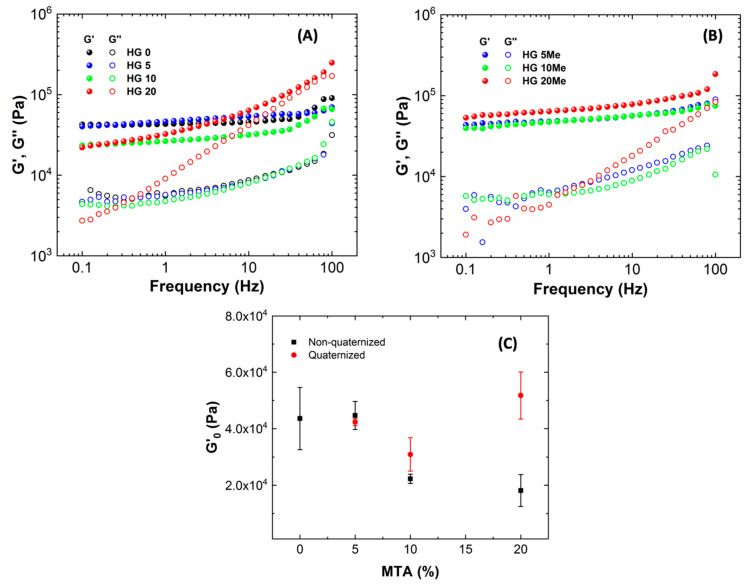
The storage and loss (G′ and G″) moduli as a function of frequency (G′ solid and G″ open symbols) for (**A**) unmodified, (**B**) methylated hydrogels and (**C**) describes the evolution of elastic modulus plateau (G′_0_) as a function of 2-(4-methylthiazol-5-yl)ethyl methacrylate (MTA) percentage for non-quaternized and quaternized hydrogels.

Figure 5A displays the storage (G‘) and loss (G‘‘) moduli as a function of frequency for all hydrogels. In all the cases, the rheological behavior is consistent with a gel, describing G‘ modulus higher than G‘‘ modulus along the whole frequency range and the storage modulus, G’, independent of the frequency. 

It is clearly observed that HG0 behavior is practically unchanged with frequency and similar behavior happens with the incorporation of small amount of MTA, HG5. However, with the incorporation of higher proportion of MTA, the G’ modulus slightly decreases as it can be seen in Figure 5C. This small diminishment is due to the slightly differences of crosslinking agent between hydrogels, as seen in Table 1. As for the quaternized hydrogels, the methylation reaction does not change much the rheological behavior of hydrogels, showing a gel-like behavior as described in Figure 5B). When analyzing unmodified and modified hydrogels (quaternized hydrogels) (Figure 5C), it can be observed how the increase of MTA gives rise to hydrogels with higher storage moduli for methylated hydrogels compared to their unmodified counterparts. As seen from the graph, HG10Me and HG20Me hydrogels show higher storage moduli G’ than HG10 and HG20, respectively, besides achieving the G’ values of HG0. This effect of modification of the viscoelastic properties could also be due to the increase of hydrophilic character that derives in higher swelling ability, as mentioned before. 

**Figure 6:**
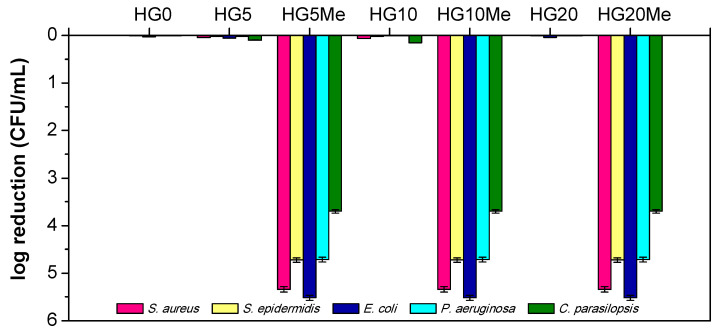
The antimicrobial ability of unmodified and methylated hydrogels against different microorganisms.

After the physico-chemical studies of the hydrogels, their antimicrobial behavior was determined by contact killing assay against Gram-positive and Gram-negative bacteria and fungi. This method directly relates the survival of microorganism (CFU mL^−1^) to the antimicrobial properties of a sample after a predetermined contact time, i.e., 24 h. Following the method, Figure 6 displays the logarithmic reduction of microorganisms with respect to a control sample, that is, without hydrogel.

As is clearly observed in the Figure, the unmodified hydrogels do not present antimicrobial activity; the microorganism population reduction is negligible. Conversely, the quaternization causes the complete elimination of bacteria and fungi. Remarkably, the methylation of the lowest composition of MTA, HG5Me, is enough to effectively kill all the microorganisms tested. Previous studies performed in contact active antimicrobial films with polymers bearing thiazolium groups (MTAs derivatives) incorporated by blending process, showed high antimicrobial efficacy when higher content were employed, c.a 30 wt% [29,30,43]. In this case, hydrogel structures seem to be suitable environments to interact with microbial cells allowing a close contact, and then higher antimicrobial activity. 

## 4. Conclusions

A series of hydrogels were prepared by combination of HEMA and MTA monomers in three different compositions, 5, 10 and 20 wt%, using PEGDA as crosslinking agent (ratio of 5 with respect to HEMA) and 2 wt% of ACPA as initiator in the thermally initiated radical polymerization at 60 °C in water solution. The posterior quaternization reaction of the thiazole groups of MTA units with methyl iodide conducts to the preparation of cationic hydrogel with potential antimicrobial activity. The quaternization reaction, which leads to the formation of cationic thiazolium groups, decreases the thermal stability of the hydrogels and enhances their swelling capacity. The viscoelastic properties of unmodified (HG0-HG20) and modified hydrogels (HG5Me-HG20Me) depict a gel-like behavior for all hydrogel compositions. Additionally, from the rheological characterization, the slight increase of viscoelastic properties for HG10Me and HG20Me compared to the unmodified hydrogels is observed, which could also be due to the quaternization. Remarkably, all the prepared cationic hydrogels independently of the content of MTA exhibit excellent antimicrobial activity against the tested Gram-positive and Gram-negative bacteria and fungi.

## Figures and Tables

**Table 1:** The weight percentage of chemicals used for the hydrogels preparation.

Hydrogels	HEMA	PEGDA	ACPA	MTA
HG0	81.70	16.34	1.96	0
HG5	78.49	15.70	1.88	3.92
HG10	75.53	15.11	1.81	7.55
HG20	70.2	14.01	1.69	14.1

**Table 2:** The characteristic parameters of hydrogels’ thermal degradation.

Hydrogels	*T*_0_ (°C)	*T*_max1_ (°C)	*T*_max2_ (°C)	Residue (%)
HG0	317		388	4.7
HG5 HG5Me	317 287	336 * 342	416 416	7.6 7.3
HG10 HG10Me	305 269	335 * 314	418 409	5.5 11.1
HG20 HG20Me	305 250	334 296	418 400	2.5 12.2

* Shoulder. Temperature standard error: ± 1 °C.
